# Accelerating implementation of adolescent digital health prevention programs: analysis of insights from Australian stakeholders

**DOI:** 10.3389/fpubh.2024.1389739

**Published:** 2024-05-03

**Authors:** Rebecca Raeside, Allyson Todd, Kyra A. Sim, Melissa Kang, Seema Mihrshahi, Lauren A. Gardner, Katrina E. Champion, John Skinner, Liliana Laranjo, Katharine Steinbeck, Julie Redfern, Stephanie R. Partridge

**Affiliations:** ^1^Susan Wakil School of Nursing and Midwifery, Faculty of Medicine and Health, The University of Sydney, Sydney, NSW, Australia; ^2^Metabolism & Obesity Service, Sydney Local Health District, Sydney, NSW, Australia; ^3^Charles Perkins Centre, The University of Sydney, Sydney, NSW, Australia; ^4^General Practice Clinical School, Sydney Medical School, Faculty of Medicine and Health, The University of Sydney, Sydney, NSW, Australia; ^5^Department of Health Sciences, Faculty of Medicine, Health and Human Sciences, Macquarie University, Sydney, NSW, Australia; ^6^The Matilda Centre for Research in Mental Health and Substance Use, The University of Sydney, Sydney, NSW, Australia; ^7^Djurali Centre for Aboriginal and Torres Strait Islander Health Research, Heart Research Institute, Sydney, NSW, Australia; ^8^Westmead Applied Research Centre, Faculty of Medicine and Health, The University of Sydney, Westmead, NSW, Australia; ^9^Specialty of Child and Adolescent Health, Westmead Clinical School, Faculty of Medicine and Health, The University of Sydney, Westmead, NSW, Australia; ^10^The George Institute for Global Health, University of New South Wales, Sydney, NSW, Australia; ^11^Engagement and Co-Design Research Hub, School of Health Sciences, Faculty of Medicine and Health, The University of Sydney, Sydney, NSW, Australia

**Keywords:** implementation science, digital health, adolescents, prevention, qualitative study, public health

## Abstract

**Background:**

Chronic disease risk factors are increasing amongst adolescents, globally. Digital health prevention programs, which provide education and information to reduce chronic disease risk factors need to be equitable and accessible for all. For their success, multiple highly engaged stakeholders should be involved in development and implementation. This study aimed to evaluate stakeholders’ support for, and perspectives on potential public health impact of digital health prevention programs for adolescents and potential pathways for future implementation.

**Methods:**

Qualitative semi-structured online interviews with stakeholders. Stakeholder mapping identified key individuals, groups and organizations across Australia that may influence the implementation of digital health prevention programs for adolescents. Recorded and transcribed interviews were analyzed within the Reach, Effectiveness, Adoption, Implementation and Maintenance (RE-AIM) Framework, using deductive content analysis.

**Findings:**

Nineteen interviews were conducted in 2023 with stakeholders from government, health, non-government organizations, youth services, education, community settings and others. Four overarching themes were identified: (i) existing digital health initiatives are not fit for purpose; (ii) the co-creation of digital health prevention programs is critical for successful implementation; (iii) digital health prevention programs must address equity and the unique challenges raised by technology and; (iv) system level factors must be addressed.

**Interpretation:**

Stakeholders broadly supported digital health prevention programs, yet raised unique insights to ensure that future programs create public health impact by improving chronic disease risk factors among adolescents. These insights can be applied in future development of digital health prevention programs for adolescents to strengthen widespread implementation.

## Introduction

Globally, adolescents 10–24 years face multiple challenges which often hinder them to live fulfilling and productive lives as adults (1). Among adolescents 11–17 years, there has been a three-fold increase in the prevalence of four or more chronic disease risk factors, such as physical inactivity and insufficient fruit and vegetable consumption (2). As such, adolescents often enter young adulthood at a higher risk of chronic diseases than when they entered adolescence (3). The roll-out of preventive health strategies that are equitable for all is one of the many priority areas for action that the Australian Government has set to improve chronic disease risk factors among adolescents (4), and this has been mirrored in global frameworks (5). To serve all adolescents who are seeking support, health care systems will require services that can overcome existing barriers that adolescents face in accessing health care services (6, 7).

The use of digital health programs to provide adolescents with education and information to reduce chronic disease risk factors is promising (8, 9). The Australian National Preventive Health Strategy has outlined that governments and health care systems should embrace the digital revolution to deliver preventive health care (10). Digital health programs are recommended to support adolescents to prevent obesity (11) and mental health prevention and treatment (12). However, programs that target mental health often do not focus on risk factors including nutrition and physical activity (13), and programs with an obesity prevention lens can be stigmatizing (14). Consultation with adolescent consumers have highlighted their desire for a holistic and integrated approach to support their health (15). Multiple stakeholders should come together to develop and implement digital health prevention programs that are both effective and in-line with adolescents needs, priorities, views, and values.

Typically, implementation research occurs after research has demonstrated effectiveness. Yet, when it comes to adolescent digital health programs, this phase-based model is potentially delaying their implementation into health care systems and community services (16). This can be for a multitude of reasons, including limited capacity and support from stakeholders, technology innovations or increased adolescent expectations for the programs (16). Engaging stakeholders meaningfully in the research process is recognized as an important strategy to translate research into public health policy and practice (17).Therefore, to implement digital health prevention programs into health care systems and community services, research is needed to map the stakeholders involved, understand their support for these programs and engage them early in the research process. The aim of this study was to evaluate stakeholders’ support for, and perspectives on potential public health impact of digital health prevention programs for adolescents and potential pathways for future implementation.

## Methods

### Study design

Qualitative study using semi-structured interviews to evaluate stakeholders’ perspectives. The study adhered to the consolidated criteria for reporting qualitative research (COREQ) guidelines (Appendix 1). Ethics approval was obtained from The University of Sydney Human Research Ethics Committee (approval number 2022/778), and all participants provided informed e-consent prior to participation.

### Participants

Participants were stakeholders identified through a stakeholder mapping process led by three members of the research team (RR, AT, SRP), which identified key individuals, groups and organizations across Australia that may influence the success of implementation of digital health prevention programs for adolescents. Following the WHO Health Service Planning and Policy-Making Toolkit (18), RR, AT, and SRP conducted a brainstorming session to identify key individuals, groups, and organizations to interview based on their experience and networks within the fields of adolescent health, digital health, and public health. For groups and organizations, websites were searched to identify the key individual(s) to invite for an interview. If unable to deduce from the website, an email was sent to the generic inbox. Stakeholders were identified across sectors including government, heath, education, industry, non-government organizations (NGOs), youth services and community groups. Stakeholders were eligible to take part if they: (i) were aged 18 years or over; (ii) had an interest in supporting adolescent populations in their sector; (iii) were willing to provide insights from their involvement in adolescent-specific digital health prevention programs; and (iv) provided informed e-consent. Once stakeholders were identified, the research team reviewed the list excluded any individuals, groups or organizations which were duplicates.

### Recruitment

Participants were invited via email to take part in an individual interview. Email invitations were sent with a link to the participant information sheet. All participants read the information sheet online, provided informed e-consent and were directed to an online survey to complete demographic characteristics (age, gender, ethnic background, sector, and location). Participants were contacted via email to organize a suitable date and time for the interview and were emailed the secure teleconference link.

### Data collection

The semi-structured interview guide was developed by the research team to address the research aims. Due to the varying nature of stakeholders, two interview guides were developed. One was developed for health and education organizations and the second for youth advocacy groups. Interviews started by the interviewer introducing the project. ‘Digital health prevention programs’ were defined as using range of technologies to protect, promote and sustain the populations health. ‘Prevention’ was defined as decreasing the risk, chance or likelihood of an individual developing chronic disease (e.g., obesity) or mental illness. Next, the interviewer asked participants broadly about digital health, their thoughts on important health prevention messages for adolescents and knowledge of any current digital or in-person health prevention programs. Following this, interview questions were structured within the RE-AIM Framework with specific questions about reach, effectiveness, adoption, implementation, and maintenance of digital health prevention programs. The RE-AIM Framework was developed to guide the planning and evaluation of programs that may assist the adoption and implementation of these into a range of settings (including health care and community settings) (19, 20).

All interviews were conducted online using Zoom videoconferencing (Zoom Video Communications Inc., San Jose, CA) from February to August 2023. Interviews were conducted by one of two female postgraduate researchers (RR and AT). Both RR and AT had previous experience in conducting interviews. The first stakeholder interview was reviewed by both interviewers to check for consistency between interview styles, no major changes were required. All participants were reminded of their ability to withdraw and that any question could be skipped if they were unsure or unwilling to answer. Interview recordings were a maximum of 45 min. Videoconference software provided separate audio and video recordings of the interviews. Video recordings were deleted, and audio recordings were retained for transcription. Transcripts were not returned to participants for comment. The semi-structured interview guide is provided in Appendix 2.

### Analysis

Participant characteristics are summarized including age, gender, ethnic background, sector of work and location of current organization. A deductive content analysis was used to analyze the interview transcripts within the RE-AIM Framework for implementation (19–21). All qualitative analysis was conducted in NVivo (NVivo 1.7). One researcher (RR) set up the coding framework with five domains: reach, effectiveness, adoption, implementation, and maintenance. All 19 transcripts were coded to the coding framework, each transcript was coded at least twice by the research team (RR, AT, SRP). Next, each category of the framework was examined by RR, AT, and SRP to identify patterns in the data and new insights were formed. Through discussions, a consensus on the underlying themes within the framework was agreed upon and those results are presented. All quotes included in the findings are from the 19 interviews.

## Results

The research team identified 60 unique stakeholders across different sectors. After reviewing the list of stakeholders for duplicates, nine were identified and removed. Therefore, 51 unique stakeholders were invited to participate in an interview. Of the 51 stakeholders invited, 19 participants were eligible and willing to take part in an interview and signed e-consent. After e-consent was signed, interviews were scheduled.

### Participant characteristics

**Figure 1:**
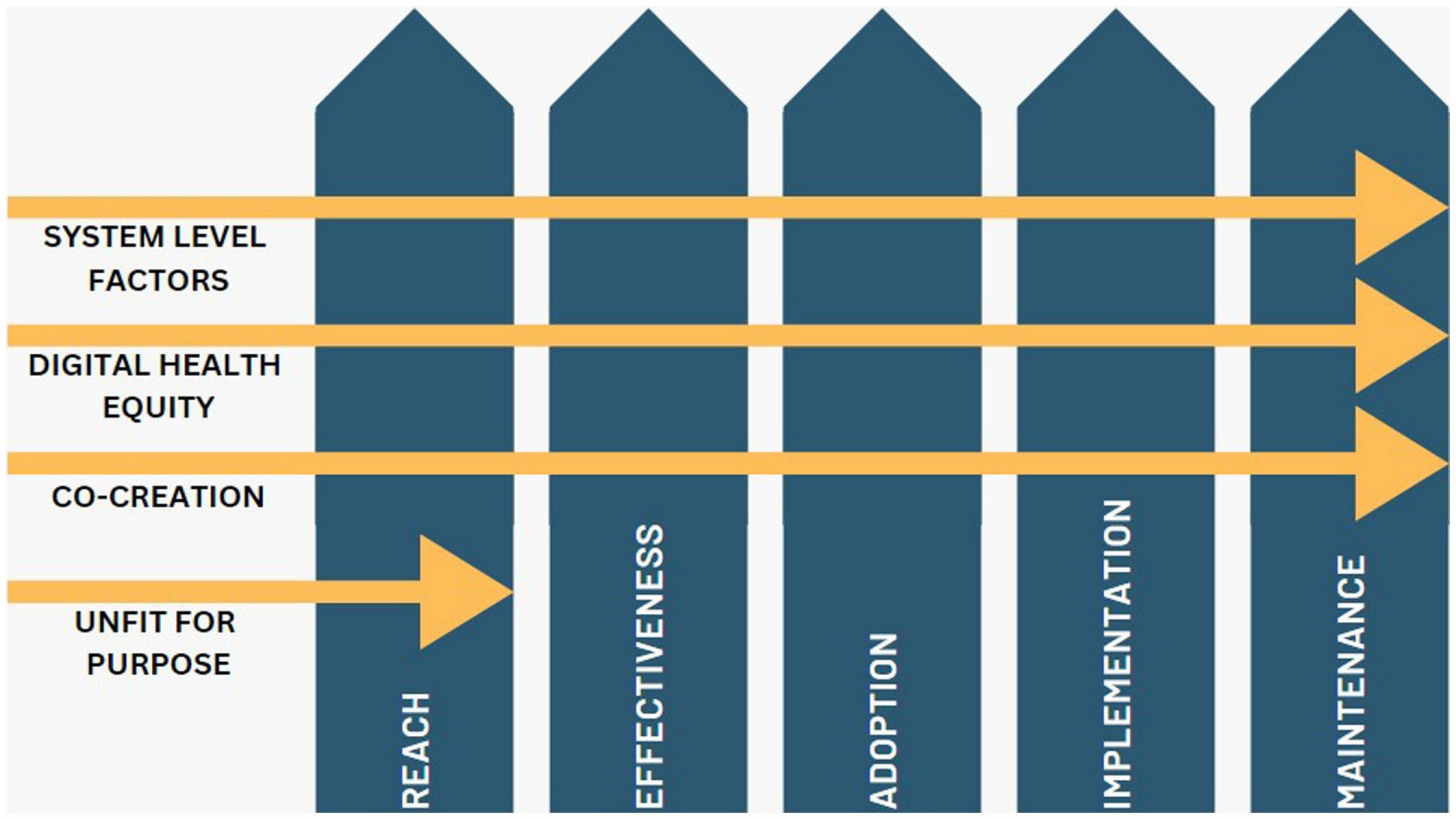
Representation of identified themes within the RE-AIM Framework for implementation.

Participant characteristics are reported in Table 1. Participants were a range of ages, with at least one in each age category (range: 18–69). They were predominantly identified as female (14/19, 74%), Caucasian (11/19, 58%) and their organization was based in New South Wales (14/19, 74%). Participants came from sectors including government (*n* = 3), health (*n* = 2), non-government organizations (*n* = 4), education (*n* = 3), youth service (*n* = 2), community (*n* = 2) or other (*n* = 3) including two adolescent health researchers and one educational designer. There was no difference in the perspectives given from different sectors, all quotes are accompanied by the sector which the participant was from. A summary of the identified themes and how they fit within the RE-AIM Framework can be seen in Figure 1, supporting quotes can be seen in Table 2.

**Table 1:** Participant characteristics (*n* = 19).

**Age (years)**	18–29	4
	30–39	4
	40–49	5
	50–59	4
	60–69	2
**Gender**	Male	4
	Female	14
	Non-binary/gender diverse	1
**Ethnic background**	Caucasian	11
	New Zealander or Maori	1
	Chinese	1
	Middle Eastern	1
	Other	4
	Prefer not to say	1
**Sector**	Government	3
	Health	2
	Non-Government Organization	4
	Education	3
	Youth Service	2
	Community	2
	Other	3
**Location of organization**	NSW	14
	VIC	4
	ACT	1

ACT, Australian Capital Territory; NSW, New South Wales; VIC, Victoria.

**Table 2:** Supporting quotes for themes identified from stakeholder interviews.

**Existing initiatives are not fit for purpose**
*‘Um, and I think there’s little bits and pieces scattered in programs like reach out and stuff like that and mental health support also.’* – ID 13 (Government)
*‘Um, so we have, we have like healthy eating programs just for young people who want to improve their nutrition. Sometimes it’s incorporated with study groups as well, just so it’s not food focused.’* – ID 16 (Youth Service)
*‘I would say that the main one would be anything that’s through beyondblue… So pretty much mental health. That will be the main one that I would know of.’* – ID 15 (Education)
*‘Yeah, it’s definitely one, I think, a strategy that can reach a lot more youth. Yeah, I guess it’s just how sort of just how you pitch it and how you reach those youth? You know, I think that’s always sometimes the hardest aspect and sort of ensuring that that you get, I guess, key stakeholders on board to sort of feed that through, as well to them.’* – ID 9 (Government)
*‘Yes, yes. There aren’t many complimentary to the little that is happening. Yes. For adolescents. Yeah. I think we, that is something that we must offer’* – ID 2 (Health)
*‘I think there would be because I think, especially, you know, I think digital health can probably be seen as being cheaper to deliver and to target a much larger group of people than other types of health prevention programs.’* – ID 6 (Other)
*‘Yeah, I think anything that can raise awareness of it. So whether it’s training staff or, you know, training adults or other demographics as well, because then that way, they can push it through different channels and different streams and advocate for it’* – ID 12 (NGO)
*‘Um certainly making teachers aware that these resources are available, and that they can possibly be integrated into teaching and learning programs not to be the way that students learn about that issue. But certainly, always, we are looking for students to develop the skills, have that health literacy and know where to go to get help. So by allowing, get letting teachers know what’s available, and what’s out there, then they can be saying, you know, putting those giving them information and say this is out there.’* – ID 15 (Education)
*‘And then I think, I mean, depending on on how, how it was implemented, and what role someone within the school had to play, it’s really about upskilling them on being able to, so if the, if there was complementary programs within the school, so through curriculum, for instance, then you’d be training up the teachers on how to implement those that teaching and learning effectively.’* – ID 14 (Education)
*‘So how are you going to engage them if they do not really care about it for now, right?’* – ID 20 (Community)
*‘and that’s I think a little bit harder in some ways to get kids to engage in because most kids do not want to engage in something unless they think they need it.’* – ID 17 (Education)
*‘So sort of seeking out those people who may need a program and then targeting something. That’s how marketing works. So if health care services or health promotion services need to be competitive in the same way that the market is they need to use the same sort of strategies.’* – ID 4 (Government)
**Co-creation of programs with adolescents is critical for success**
*‘So maybe incorporating that sort of thing where they can add their own friends, but also connect with other people as much about it.’* – ID 6 (Other)
*‘I think the easiest way to work with them is not to try and go into those communities and run your own workshop, but rather you connect with those communities first and you find a community member that is, that is placed on formal leadership, right.’* – ID 20 (Community)
*‘and also, if there’s things that are made clear, like if it was inclusive of all young people, if they were … interpreter services available, if, you know, they felt like and I think they felt that there was transparency, and if people could give feedback, and they can see how what other people’s experience with this services have been? …. So I feel like if these conditions aren’t met, young people would not do that.’* – ID 10 (NGO)
‘*Yeah, and as you know, language changes even like what’s what interests kids and where it’s cool to be, and what platforms it’s cool to be on is constantly changing. Like, we cannot keep up*’ – ID 17 (Education)
*‘And how do you maintain that target audience? Especially as that target audience is kind of like shifting? How do you get them in once they are kind of spitting themselves out at the end? And then where do they go from there?*’ – ID 12 (NGO)
*‘Um, the biggest barriers would be I guess. Like I said, it, you are competing against so many other things that are online? So yeah, I think distraction does not sound like a very real barrier. But I feel like that’s a big barrier for young people just actually getting onto their phone and using it’ –* ID 16 (Youth Service)
*‘I think having good engagement from adolescents themselves to help design the intervention, so that you sort of designing something that you know, is going to be appealing to them.’* – ID 6 (Other)
*‘I think the health professionals understanding and the young people’s perspective is quite different. Some, often, health professionals think that young people just will not engage in content, whereas really, there’s a lot of engaging content out there. Young people are more focused on what they can trust. So I think perhaps, that’s shifted.’* – ID 4 (Government)
**Digital health equity and unique technology challenges**
*‘I think a lot, even if you do live regionally or rurally, I think most youth these days have a phone. Yeah, I think yeah, I think a lot. I think it’s definitely probably one of the … a key strategy for accessibility.’* – ID 9 (Government)
*‘So it’s got to be a safe and well respected at the same time as being fun, edgy, different, changing all the time.’* – ID 2 (Health)
*‘access to devices, access to you know, whether it’s phones Wi Fi computers, you know, like the like, we take, I think, there you go, it’s easy to take that connectivity for granted. There are a lot of different groups of young people, particularly socially excluded groups, young people, that’s not a given.’* – ID 7 (NGO)
*‘But then I think another thing, it, we have to remember is it cannot be used as a substitute for things that cannot, you know, be digitalized.’* – ID 10 (NGO)
‘*young people have been yelling out for more like innovative supports around health’* – ID 13 (Government)
*‘Especially for younger people, they feel a lot more comfortable doing things online. I cannot say that this is the same for people that are not fluent in English, and that are not digitally literate. But for young people. There’s definitely an element of relief, a lot of the time when they know that they can access a service without having to go out, they can just pick up their phone or their computer and do something about it. Because it can be a lot less daunting.*’ – ID 10 (NGO)
*‘And now, you know, making that digitally accessible, can help in a lot of ways, but can create other barriers. So like I said, I think it’ll be helpful to have different languages available and making it clear that this is like an inclusive space for people with different, different ethnicities, different sexualities’* – ID 10 (NGO)
*‘I mean, I think health is and well being it’s sort of a private journey, in a way. So I guess that’s the benefit that I can see that people can explore topics, and it might, you know, an information that that is what they need right now without necessarily going into a clinic or, you know, that sort of thing.*’ – ID 8 (NGO)
*‘I think, you know, some interaction with platforms that they are already using. It would be helpful. I do not know if that’s necessarily possible. But it’s, I think that’s a way to improving engagement, if it can link in with, with things that they are already doing.’* – ID 6 (Other)
**System level factors**
*‘Monkey bureaucracies, slowness, rigidity, inflexibility, lack of imagination, people who are barriers who have never been involved…’* – ID 2 (Health)
*‘It’s harder for those to work these days because eyeballs are so distributed across different platforms and the algorithm means you do not always see the same stuff.’ – ID 18 (Other)*
*‘Well, I think you, well, even within a New South Wales Health program, there’s no one to refer it to, because they are all overwhelmed. Like, like, quite honestly, there aren’t enough services’* – ID 2 (Health)
*‘So I guess the answer would be like, it has to be constantly, like the impact has to be evaluated constantly. And there has to be a way to be able to see if it’s making a change, you know, to know if anything has to be updated, or stopped at all.’* – ID 10 (NGO)
*‘And so if it’s kind of just info, being spat at them, it just will not work. They just, they really need things to kind of intrigue them or you know, even if it is, I do not know, it, even if it is something some information that you want them to know, the way that it’s drawn across to them has to be interesting for them to even click on it sometimes. But I would say interactive is always going to be the best way.’* – ID 5 (Health)
*‘So having a I guess like a support system behind that or you know somebody who can be a point of call. So if that young people do have questions that you have someone you can refer them to… So we can talk to and we can check in with them to see how the young person is going. And if from our side, we, when we are chatting or catching up with young people, we heard something concerning around the services, we can always directly send feedback to the services as well.*’ – ID 19 (Youth service)
*‘I think it’s helpful for it to be linked with some sort of platform that goes forever. Because I think ultimately, what is successful on social media is repeat messaging. And I do not know how successful we are with sort of having a message given to them one or two times, and then having that implemented over the long term, I think really, you need to just have that repeat messaging. So I would almost think a very short, pro actual program, like a formal program would be quite short. But then the they would have access to content forever.’* – ID 6 (Other)
*‘their interest change very rapidly, like they have a focus on something like it’s the thing of the live class for 3 weeks, and then it goes into something else. So it may be that then, if the program is for, as long as 6 months, you might need to have a bit of dynamic’* – ID 3 (Other)
*‘I suppose your issue is how can we do this as cost effective a way as possible, get maximum effect for the resourcing that we have. And if you can deal with a whole range of other if there’s a range of other messages that are being done at the same time, rather than this is the eating disorder group. This is the alcohol group. This is a smoking group. This is the healthy lifestyles group. Maybe it would be better if those things were actually, if there was resourcing for all of them.*’ – ID 2 (Health)
*‘But then it’s how do you get people to use that. And if that that mix of you need to have something that looks engaging, speaks their sort of language, you probably need to look at some sort of initial marketing, and promotions, or media type launch, to probably a mix of all really like you can do a media launch, and it gets 1 day of coverage. But you really need that sort of follow up social media sort of amplification as well.’* – ID 11 (Youth Service)
*‘Yeah, and as you know, language changes even like what’s what interests kids and where it’s cool to be, and what platforms it’s cool to be on is constantly changing. Like, we cannot keep up. We can, we can think we are on top of it here. And we know where they get hit with, like things like online bullying happening or whatever. But the next thing is, there’s a whole new platform for them to work from that were totally unaware of. So I think there is a need to, to, to update relatively often, I’ve had so much update, but check relevance. And check it against your test. Who you are trying to get who you are trying to reach.’* – ID 17 (Education)

### Themes

#### Existing digital health initiatives are not fit for purpose

When asked about knowledge and reach of current digital health prevention programs for adolescents, many participants could not recall current examples of existing programs unless their role was situated in a school setting. Other programs which were commonly identified were mental health programs (e.g., headspace, Beyond Blue) yet they were unsure of the level of preventive health information within them.

*‘I know of pilots of things, but not nothing that’s sort of been picked up and run. And I’m sure there are ones. In fact, I’m absolutely sure there are ones in the sort of mental health space, but I’m not so much. I’m not really an expert on those. So I do not, I’m not so aware of that.’* – ID 2 (Health)

*‘I’m not aware of any sort of public health type initiatives other than what’s delivered in schools. So like I know about the school programs, they obviously look at health and nutrition and physical activity and mental health as part of the school curriculum. In primary school and in high school, but I’m not aware of any sort of broader public health programs.’* – ID 6 (Other)

When asked about the potential of future programs, participants showed broad support for the development and implementation of digital health prevention programs. They also suggested that they should be complementary to existing face-to-face initiatives.

*‘I think there’s the potential to reach a lot of adolescents because we know that they are all online. Almost all of them are online and using social media. And so I think there is the potential to do it.’* – ID 6 (Other)

*‘Yeah, yeah, definitely could be complimentary. A lot of it, though, depends, I suppose on uptake and the willingness of young person to act to actually engage.’* – ID 15 (Education)

However, digital health prevention programs were not considered as a stand-alone setting. All stakeholders considered that programs would sit within existing initiatives, including school curriculum or complementary programs. However, these are constrained by limited resources and existing structures which have been shown to be ineffective. Specific contextual factors that were raised within this were the appropriate use of devices within a school setting and raising awareness of programs with staff.

*‘I do not see the digital health and sort of taking over it simply that it’s going to be people are made aware of it. And so therefore, kids can be, you know, sort of directed to it as an extra source of help and support’* – ID 15 (Education)

*‘So for some people, digital health services can be the right level of support to address their problem, or the right mode. And that’s it. But for others, it’ll be sort of an adjunctive to support to other sort of perhaps clinical in person service delivery, that they are accessing as well. So it really like the sort of market is sort of almost endless, really, because there are so few young people who are not digitally engaged.’* – ID 11 (Youth Service)

*‘I think, what we one of the things, though, that we hear from young people is that younger people still relational. So there’s, all of those things are good, and they like having them as an option. But I do not want them to replace face to face connection with, you know, workers or doctors or health professionals or, you know, whoever, you know, whoever would otherwise have ran a program before it became an online thing.’* – ID 7 (NGO)

Another consideration raised was that a chronic disease prevention lens is not engaging for young people as it is not necessarily useful to them, and future programs will need to go through a different lens, e.g., holistic view of health and wellbeing.

*‘I mean, the difficulty of prevention is that for people to uptake, the intervention I guess it necessitates that they have a sense of usefulness for them. Like why would they so being proactive about their health? And I’m not sure all young people are really conscious or aware of that.’* – ID 3 (Other)

*‘So I think for them, it’s more wellbeing is a really holistic concept …it’s about how you feel mentally, it’s about the environment that you live in. It’s about whether you, you know, how you feel within yourself … I think for young people, it’s much more holistic than it is for adults, whereas adults, it might be about, here’s how you prevent diabetes, and here’s how you prevent heart disease. And here’s how you prevent, I do not think young people think in those terms, I think much more broadly about just how I feel about myself.’* – ID 7 (NGO)

Finally, it was recognized that it would be important to choose the right contact points to introduce a future digital health prevention program to adolescents. It was suggested that multiple entry points would be needed to engage adolescents. The two entry points which were identified and supported most were through schools and social media.

*‘I think there’s no one way to reach out to young people and to help them engage, they have different styles, different types of things they like’* – ID 3 (Other)

*‘Um, it really depends on what I mean, if you are talking about physical activity, healthy eating, I would say probably best through schools’* – ID 5 (Health).

*‘or it could be like social media. And perhaps, to get over that sort of hurdle of signing up, it might be good to engage some sort of influencers of potentially to talk about and encourage signing up.’* – ID 11 (Youth Service)

Since current initiatives are not fit for purpose, this theme did not extend beyond the exploration of reach within the RE-AIM Framework. There were three further themes which were identified by participants to be critical for the successful implementation of future digital health prevention programs for adolescents, which were identified within all five categories of the RE-AIM Framework and will be expanded below.

#### Co-creation of programs with adolescents is critical for success

Firstly, participants identified that future digital health prevention programs need to be co-created with a diverse group of young people for them to be supported by their organizations. They also had specific considerations including that programs were evidence-based, relevant to adolescents and target them directly.

*‘I think if it’s, if it’s sort of, if it’s based on evidence, like evidence-based action is really key to us and youth informed, you know, youth, genuine youth engagement and co-design. If those two things are in place, then it’s certainly something we’d support.’* – ID 8 (NGO)

*‘And, you know, we would have to make sure that it’s even relevant to them at all. Like, we cannot just share it with them. And then they are like, well, I cannot read this. You know, it’s not I cannot, I’m like, I’m not fluent in this language. So what’s the point? Why did you say that? So I think it’s definitely depend on all of this.’* – ID 10 (NGO)

Co-creation of programs was also viewed as a driver of acceptability and engagement with programs. The inclusion of peer leaders or champions was suggested to help drive program engagement, particularly for diverse communities. Furthermore, working through already trusted networks was seen as vital for future programs to be acceptable to adolescents.

*‘So it would have to feel like this was made, you know, in informed by people just like them? I think. So it’s all again, going back into this is like, really, like you cannot make a service for adolescents without including them in the design in some way. And it cannot be tokenistic. Like they have to feel it in the way that they are seeing what it looks like, but the service looks like and for them to even learn about it.’* – ID 10 (NGO)

*‘…and so I guess, if they have already got a relationship of trust with that person, whether it’s their peers, young people, you know, their peers, or the doctors or their parents. I think, I think the having someone that’s reliable and trustworthy is probably key.’ –* ID 8 (NGO)

Two barriers were identified to the successful implementation of digital health prevention programs at an individual level. Firstly, the balance in language and imagery to accommodate all diverse young people would need to be just right for them to engage with a digital health prevention program. The second barrier is competition with what is already available at schools and on social media.

*‘So if you can make it as interactive as possible, I would say that’s the best thing. Yeah, the barrier would be too much or too little info. It’s trying to get that right kind of nice size, nice imagery, nice colors across for them to really be like, oh, this is quite cool.’* – ID 5 (Health)

*‘But the biggest challenge, I think, is just going to be competing with what’s already out there and trying to come up with a delivery approach that is engaging enough for them.’* – ID 6 (Other)

The ongoing evaluation of programs was recognized by participants as essential for long-term successful implementation. Programs cannot be co-created and then left to run without evaluation, they must be dynamic to be able to accommodate any necessary changes, both in terms of the technology and the content. Individual feedback from adolescents will demonstrate the changing nature of their views and needs.

*‘And then like, obviously, you mentioned the research and evidence base is always evolving. So you have got and like young people, their service expectations, and where they are at in terms of the issues they are facing, or their approach to things that’s evolving as well. So you have got like, all of these sort of cycles working at once, which is sort of kind of unique to the digital sort of space.*’ – ID 11 (Youth Service)

By co-creating digital health prevention programs with young people and constantly evaluating them it may help to overcome the barriers identified and assist with the long-term successful implementation.

*‘If you if you want to target them, I think it’s about like, trusting that they are the right person to inform how it should be.’* – ID 10 (NGO)

#### Digital health equity and unique technology challenges

Participants raised concerns around the equity of digital health prevention programs. This came from an access point of view, with the concern that adolescents who are most in need of these programs will not engage (e.g., rural and remote residing, culturally and linguistically diverse [CALD] populations). However, it also extended to incorporate the safety and trustworthiness of future programs.

*‘I guess there are those kind of equity issues around, you know, rural, regional, and just different environments, and how, depending on which environment you are, you might be more susceptible to different mental health issues or, you know, access to food, and obesity.’* – ID 12 (NGO)

*‘You do need that massive drawing area, because what meets the needs of a kid living in a city is not going to meet the needs of the kid living in a country town or living in a regional part of Australia or, or in an isolated part of Australia’* – ID 17 (Education)

*‘But if it’s about a particular health message, they may receive it differently if it’s from someone that had that medical background, or someone that they already trusted in that sort of space.’* – ID 8 (NGO)

The accessibility of digital health prevention programs was identified as both a barrier and enabler. Being digital provides access in a modern context, especially as adolescents can be more comfortable with doing things online. However, it must be ensured that the program is accessible to all and is not widening the digital divide.

*‘Well, they are asking for it! I think the strengths of a digital health approach that is it allows health access to be more functional in the modern world. Like, it’s been a bit of a slow pull, getting the medical system to digitize and it’s just unnecessarily slow, like the whole world is technological now, just that it’s really important that we invest in upgrading the health system in that way. It allows flexibility for young people to be able to access health independent of their parents or carers, which is really important because a lot of you know, especially around like mental health and, and sexual health and drugs and stuff like that a lot of young people do not, wont access health system because they have to go through their parents to ask about those tricky subjects.’* – ID 13 (Government)

*‘I would say from a health … equity point of view and working with people low socio-economic status and from cultural, linguistically diverse communities, it’s more of whether the technology that you are putting up is suitable for the devices that they have access to, right? It’s pretty much a given that everyone has access to a mobile device these days, but it’s the how high powered that device is’* – ID 20 (Community)

When discussing implementation at an individual level, two main enablers were identified. Firstly, it was recognized that for adolescent’s health and wellbeing is a private journey, and by providing access to preventive health information online from a reputable source it may be better than online sources that they are currently accessing. Secondly, adolescents were identified as a captive audience through their presence at schools and on social media, these would cast a wide net over the adolescent population and be ideal entry points to a digital health prevention program.

*‘And I would say also, digital health prevention, prevention of preventative space is really good if there are things online that you want to ask that you do not want other people to know about. So especially what I hear through a lot of our young people is that they will go to Reddit and other apps that to source their information that’s a bit worrying in some ways that that’s where they go to so educational programs’* – ID 5 (Health)

*‘That you have got a captive audience, that’s the first thing that yeah, that there’s opportunities for, for teaching and learning to sit alongside it, that’s, you know, that they can learn about what they are doing and the outputs of, of the program.’* – ID 14 (Education)

For implementation at a setting level, the first enabler which was identified by participants was that the platform on which the program is delivered needs to be credible, innovative and in spaces that adolescents already engage with. Secondly, it was suggested that the program could be multicomponent to increase engagement and provide repeat messaging across different platforms. Finally, it needs to be flexible to adapt with the ever-changing digital world.

*‘And there’s a lot of money again, government it’s a lot of money into all kinds of digital solutions that aren’t, you know, they have they are not tested, they do not work and there’s a lot of money that went into them. And that’s, that’s terrible when we see that happening. So yeah, it’s about doing all of that, that kind of piloting and testing first. And then once it’s right then making the commitment to make it sustainable.’* – ID 7 (NGO)

*‘Yeah. It’s hard because the digital world changes so quickly, and what’s cool and trendy one day, and I mean, even possibly looking at this is a thing that embedding it in some of the things that kids commonly use, so messages that come through on Instagram or, or Snapchat…’* – ID 17 (Education)

#### System level factors

System level factors extends to the technology systems which deliver the program, the health system and current available services and other resources which may be needed for successful implementation of digital health prevention programs. Inflexibility of the platform was one of the setting level implementation barriers identified by participants. It was acknowledged that whatever platform used would need to be able to keep up with technology changes. Furthermore, some participants raised the issue of bureaucracies that exist within government and the health system which may hinder future partnerships and implementation of digital health prevention programs.

*‘The other thing that kids I find increasingly used to the laptops as well. So just that access on across platforms, I guess, the phone, but also kids will get online and like say I suppose the app though, I suppose the phone is the app level. So that’s a strength in that you can put it there, but you can also have I suppose computer compatible and proper technology, computer compatible programs that they can access from that level too.’* – ID 17 (Education)

*‘So if you want, for example, [xxx] to be, to have their logo and be a part of that process to lend their like respectability and, and reputation to the process that can be a long winded, very bureaucratic process. It’s very resource heavy.’* – ID 13 (Government)

Another implementation barrier which was identified at a setting level is that the health system is not oriented toward adolescents, considering there are not enough services available for them and the services which are available may not suit their needs. Finally, the resourcing needed to run a digital health prevention program for adolescents was seen as a barrier to successful implementation.

*‘And a lot of young people are often transparent to the health system, the health system is not oriented toward them, will not put the effort into them, and will not bother to speak with them deliver the services for them, actually put a bit of effort into getting something that will appeal to them.’* – ID 2 (Health)

*‘I think you just need it needs to be adequately funded to be able to pay for advertising and have the staffing who can you know, create new content regularly to keep it relevant. I think that’s going to be the biggest challenge.’* – ID 6 (Other)

*‘And I’m like the outlier if it’s a always on* versus *a supported, like person behind service or not sort of thinking about, well, what hours are young people going to access the service and it might not be nine to five, Monday to Friday.’* – ID 11 (Youth Service)

To combat these barriers several solutions were suggested by participants. The first is that constant evaluation of the program is needed. This can be through various means including through back-end data from the digital platform and individual participant feedback to assess impact and measure health outcomes.

*‘Yeah, I think like, it’s all backend stuff, right? Because you’d want to have ability, and you’d want to like if it was an app, you’d want to mimic on the things that work. So you know, the ability to like something, the amount of time that somebody’s watching the video or engagements’* – ID 12 (NGO)

*‘And then because digital moves so quickly, it could be like 6 months, or a year and the world has moved on. So I think it’s like embedding that sort of strong feedback loops, both with users themselves, and like, a sort of broader evaluation like program evaluation and outcomes.’* – ID 11 (Youth Service)

The ability of the platform to be adaptive to technology and infrastructure changes was recognized as imperative for long term implementation. Participants provided examples of the wavering engagement from adolescents when the program was not flexible.

*‘Oh well, they probably used it for a couple of years and the main drop off was again it comes to those design things, people update their phones it’s they did not it was made with one-off funding so they did not have the funding to keep updating it. So what began is students started to start. It started to have bugs and those sorts of things. And it’s, you know, and so it started to be like, oh, hang on a second, only half the students seem to be able to access it, you know. And so it became something that over time it just did not have that longevity.’* – ID 18 (Other)

*‘You know, I think it would need to be reviewed at least every year or two, because technology is moving so fast that, you know, when I first started looking at social media, like Instagram was the most popular app for adolescents. And a year or two later, it was TikTok, and Snapchat. And so to remain relevant, I think you probably have to review the digital platform every year just to see where things are going.’* – ID 6 (Other)

Various resources were identified by participants as necessary if a digital health prevention program is to be implemented. Firstly, enough funding is needed to ensure that the program is continuously available and that it is delivered in a way which is cost-effective, which may take a multicomponent approach. Furthermore, there is a need to consider the demand that may be placed on other services through referral from the digital health prevention program.

*‘So if there’s going to be like action messages to the adolescent, or if there’s going to be support in, you know, if you are feeling like this, here are the services that are available that you can contact, I guess making sure that those service providers are well aware of the demand that could be created.’* – ID 12 (NGO)

*‘the biggest thing we hear from young people, and again, it’s not just in this area, but in many areas, really great programs come along, and they are awesome. And then the funding runs out, and they stop. And that’s really frustrating for young people. And it’s really difficult if it’s something that they thought was really valuable.’* – ID 7 (NGO)

For successful maintenance of digital health prevention programs, participants identified that partnerships would be necessary. This would be a potential solution to the issue above around the demand placed on other service providers. Secondly, partnerships will help not only with the launch of a future program, but also with the ongoing amplification and sustainability. This was suggested by stakeholders to occur on social media.

*‘I would think maybe a shorter program that’s only 6, 3 months or something short, that then they can link into a YouTube channel or article … or something that provides them with information and content forever, until that goes out of fashion. And then you switch to the new the new platform and whatever it is’* – ID 6 (Other)

*‘So having a I guess like a support system behind that or you know somebody who can be a point of call. So if that young people do have questions that you have someone you can refer them to… So we can talk to and we can check in with them to see how the young person is going. And if from our side, we, when we are chatting or catching up with young people, we heard something concerning around the services, we can always directly send feedback to the services as well.’* – ID 19 (Youth service).

## Discussion

This qualitative study found there was broad support from stakeholders across sectors for digital health prevention programs targeting adolescents. Using the RE-AIM framework, new insights were uncovered under four main themes: current digital health initiatives are not fit for purpose, co-creation of programs with adolescents is critical for success, digital health equity and unique technology challenges and system level factors. Stakeholders had limited knowledge of current initiatives that had a specific focus on prevention of chronic diseases and provided unique perspectives on barriers and enablers to the implementation of digital health prevention programs, and strategies to ensure their long-term success. Co-creation with adolescents was viewed by stakeholders as essential for the future development and implementation of digital health prevention programs. It was also recognized that digital health equity must be considered, along with the unique challenges that technology brings. Finally, system level factors including resources and digital infrastructure must be considered for success.

Results from global initiatives (e.g., 1point8 for change) reveal that affordable, high-quality adolescent health and wellbeing services through digital platforms are important to young people (22). Adolescents are digital natives and early adopters of technology; therefore, delivery of these services digitally is a scalable and equitable solution. It is important to recognize that digital divides exist, yet they generally mirror socio-economic divides (23). Therefore, to address equity in future digital health prevention programs, it will be important to address socio-economic factors within them. Systematic review evidence has also shown that adolescents are receptive to the use of digital health for preventive services (24). However, successful implementation of these services does not come without challenges. A scoping review aimed to uncover challenges on the use of digital health prevention programs among adolescents and found three key challenges were the disconnection between digital health and clinical preventive care, threats to the privacy and security of young people, and trouble finding valuable digital health programs for young people (25). These findings are not unique to high income countries, a systematic review found that the sustainability of digital health interventions in low- and middle-income countries is also complex and multidimensional (26). However, digital health solutions are promising in low- and middle-income countries, where mobile phones are used for internet access (27). The results from this study provide important findings from stakeholder’s perspectives to help combat some of these previously identified challenges.

A key finding from this study was that stakeholders viewed the co-creation of digital health prevention programs to be essential for their success. Though the word ‘co-design’ was commonly used by stakeholders within interviews, co-creation is a more appropriate term to describe the true meaning of their intention to involve adolescents. Co-creation refers to the collaborative nature of problem solving from problem identification through to implementation and evaluation (28). Co-creation of research has been found to improve health related outcomes among adults (29), yet limited research is available to understand how this extends to adolescent health outcomes. Adolescent engagement in research often occurs once research design and protocols are in place (30). A scoping review investigating adolescent participation in chronic disease prevention research found only 11% of studies engaged adolescents in all five stages of the research process (31). Co-creation can ensure that programs are engaging and provide value, and that they represent views from all diverse young people. However, diverse young people may have different requirements or preferences, thus it is important that the parameters of resources and funding are outlined from the start (32). By creating safe, trustworthy, and inclusive environments for adolescents to co-create solutions together with researchers and stakeholders, there is likely to be greater impact and long-term success.

Another finding from this study is that stakeholders did not consider digital health prevention programs as a stand-alone setting for delivering preventive health care, highlighting the need to address system level factors in future program development and implementation. Though the Australian health care system performs well on international standards, it has been recognized that it is too complex to navigate with multiple providers at local, state and national levels (33). This results in a system which is failing to provide equitable access to all. Though public views have improved over time, access to care is still recognized as an area for improvement (34). Recommendations from prior research have highlighted that technology should be fully utilized by health services to promote engagement as well as health care (35). The Australian school system was also considered, however up to 20% of students are consistently disengaged (36), and previous digital health preventive programs within a school setting have been unsuccessful in modifying chronic disease risk factors (37). Furthermore, a prior scoping review called for more research to successfully implement digital health programs to achieve primary prevention in settings including formal organizations such as schools and healthcare facilities, and less formal settings including households and neighborhoods (38). Stakeholders in this study broadly supported the implementation of digital health prevention programs and recognized that they are a way to provide equitable access in a modern context. Partnerships within the health care system, schools and related services are potential solutions for successful long-term implementation of digital health prevention programs for adolescents.

This study found that working through already trusted networks would be key to successful implementation of digital health prevention programs for adolescents. A WHO-UNICEF-Lancet Commission found that assurance of data privacy and security is key to digital innovations (39, 40), and reviews have suggested to prioritize ethical research which addresses data privacy (40). Adolescents are particularly vulnerable in the digital world for a multitude of reasons. They are forming online identities while their offline identities are still forming, thus their usage of technology and information sharing is potentially at risk (41). Also, digital health literacy skills of adolescents need to be considered so that they can confidently evaluate the information delivered (42). Therefore, it is imperative that digital health prevention programs address these vulnerabilities within the digital infrastructure. The co-creation of programs with adolescents and use of peer champions may assist to address this issue, so they can learn through peers that the digital health prevention program is safe and trustworthy to use.

A strength of this study is that we interviewed stakeholders across a broad range of sectors including government, health, community, education, NGOs and youth services. Though stakeholders were from a broad range of sectors, there was a small sample in some sectors and did not gain depth within some sectors which may be a limitation of the study. A further limitation is that we only interviewed stakeholders from three states and territories in Australia. Despite this, clear themes were evident in the data collected and some of the stakeholders operated at a national level and thus gave insights from across Australia. Another potential limitation to the study is that the findings are unique to the context and may not be widely generalizable beyond Australia. However, similar insights have been uncovered in other global studies (25, 26, 43). Finally, this study only demonstrates the views of stakeholders and does not represent the views of adolescents. Future research may strive to confirm these results with adolescents to understand whether they identify any additional barriers or enablers to implementation.

### Conclusion

This qualitative study found that there were broad levels of support from stakeholders for the implementation of digital health prevention programs for adolescents. Through using the RE-AIM Framework for implementation, four overarching themes were found. Firstly, existing digital health initiatives are not fit for purpose. For successful implementation of future programs, stakeholders identified that co-creation of programs with adolescents is essential, digital health equity needs to be considered and system level factors should be addressed. Research is needed to apply these insights into future program development to accelerate widespread implementation of digital health prevention programs.

## Data availability statement

The datasets presented in this article are not readily available because individual participant data will not be made available. Participant quotes can be found in the manuscript and in Table 2. The discussion guide is available as Appendix 2. Requests to access the datasets should be directed to rebecca.raeside@sydney.edu.au.

## Ethics statement

The studies involving humans were approved by The University of Sydney Human Research Ethics Committee (approval number 2022/778). The studies were conducted in accordance with the local legislation and institutional requirements. The participants provided their written informed consent to participate in this study.

## Author contributions

RR: Conceptualization, Data curation, Formal analysis, Investigation, Methodology, Project administration, Validation, Writing – original draft, Writing – review & editing. AT: Conceptualization, Data curation, Formal analysis, Methodology, Project administration, Validation, Writing – original draft, Writing – review & editing. KAS: Conceptualization, Writing – original draft, Writing – review & editing. MK: Conceptualization, Writing – original draft, Writing – review & editing. SM: Conceptualization, Writing – original draft, Writing – review & editing. LG: Conceptualization, Writing – original draft, Writing – review & editing. KEC: Conceptualization, Writing – original draft, Writing – review & editing. JS: Conceptualization, Writing – original draft, Writing – review & editing. LL: Conceptualization, Writing – original draft, Writing – review & editing. KS: Conceptualization, Writing – original draft, Writing – review & editing. JR: Conceptualization, Funding acquisition, Methodology, Supervision, Writing – original draft, Writing – review & editing. SRP: Writing – original draft, Writing – review & editing, Conceptualization, Formal analysis, Funding acquisition, Investigation, Methodology, Supervision.
