# Supplementary material for: Accelerating implementation of adolescent digital health prevention programs: analysis of insights from Australian stakeholders
Source: Front Public Health. 2024 May 3;12:1389739. doi: 10.3389/fpubh.2024.1389739 (PMC11100413; doi:10.3389/fpubh.2024.1389739)
Supplement: Supplementary file 1 [file Data_Sheet_1.PDF]

Appendix 1: Consolidated criteria for reporting qualitative research (COREQ) Checklist

**Domain 1: Research team and reflexivity**	
Personal Characteristics	
1. Interviewer/facilitator: Which author/s conducted the interview or focus group?	All interviews were conducted by RR and AT
2. Credentials: What were the researcher's credentials? E.g. PhD, MD	RR holds a MPH and BBiomedSc, AT holds a MPH and B. Global Studies
3. Occupation: What was their occupation at the time of the study?	RR is a Research Associate, AT is a Research Officer
4. Gender: Was the researcher male or female?	RR and AT both identify as female.
Experience and training	
5. What experience or training did the researcher have?	RR has organised, supervised and taken notes for >15 focus groups, led 5 focus groups and conducted >20 individual semi-structured interviews with research participants.<br><br>AT has organised, supervised and taken notes for >5 focus groups and conducted >15 individual semi-structured interviews with research participants.
Relationship with participants	
6. Relationship established: Was a relationship established prior to study commencement?	RR had no prior relationships with any of the participants who took part.<br><br>AT had a prior relationship with two of the participants.
7. Participant knowledge of the interviewer: What did the participants know about the researcher? e.g. personal goals, reasons for doing the research	None of the participants knew the interviewer prior to the interviews.<br><br>All participants read and signed e-consent forms therefore they knew the reasons for conducting the research.
8. Interviewer characteristics: What characteristics were reported about the interviewer/facilitator? e.g. Bias, assumptions, reasons and interests in the research topic	No characteristics were reported
**Domain 2: study design**	
Theoretical framework	
9. Methodological orientation and Theory: What methodological orientation was stated to underpin the study? e.g. grounded theory, discourse analysis, ethnography, phenomenology, content analysis	Deductive content analysis within the RE-AIM Framework for Implementation
Participant selection	
10. Sampling: How were participants selected? e.g. purposive, convenience, consecutive, snowball	Convenience and snowball
11. Method of approach: How were participants approached? e.g. face-to-face, telephone, mail, email	Participants were invited via email.
12. Sample size: How many participants were in the study?	51 participants were invited<br>19 interviews were conducted

13. Non-participation How many people refused to participate or dropped out? Reasons?	32 participants did not respond to the email invitation.
Setting	
14. Setting of data collection: Where was the data collected? e.g. home, clinic, workplace	Online using Zoom teleconference
15. Presence of non-participants: Was anyone else present besides the participants and researchers?	No
16. Description of sample What are the important characteristics of the sample? e.g. demographic data, date Data collection	Demographic data
17. Interview guide: Were questions, prompts, guides provided by the authors? Was it pilot tested?	Questions were asked by the interviewer but not provided to participants. Interview guide was not pilot tested.
18. Repeat interviews: Were repeat interviews carried out? If yes, how many?	Yes, 19 interviews were conducted.
19. Audio/visual recording: Did the research use audio or visual recording to collect the data?	Audio recording was used to collect data via Zoom teleconference and iPhone voice memos
20. Field notes: Were field notes made during and/or after the interview or focus group?	Yes, after.
21. Duration: What was the duration of the interviews or focus group?	45 minutes
22. Data saturation: Was data saturation discussed?	Yes
23. Transcripts returned: Were transcripts returned to participants for comment and/or correction?	No
**Domain 3: analysis and findings**	
Data analysis	
24. Number of data coders: How many data coders coded the data?	Two data coders with discrepancies checked by third independent coder
25. Description of the coding tree: Did authors provide a description of the coding tree?	Yes
26. Derivation of themes: Were themes identified in advance or derived from the data?	Derived from the data.
27. Software: What software, if applicable, was used to manage the data?	Transcripts produced in Microsoft Word, thematic analysis in NVivo (1.7)
28. Participant checking: Did participants provide feedback on the findings?	No
Reporting	
29. Quotations presented: Were participant quotations presented to illustrate the themes / findings? Was each quotation identified? e.g. participant number	Yes
30. Data and findings consistent: Was there consistency between the data presented and the findings?	Yes
31. Clarity of major themes: Were major themes clearly presented in the findings?	Yes
32. Clarity of minor themes: Is there a description of diverse cases or discussion of minor themes?	Yes
