# Supplementary material for: Accelerating implementation of adolescent digital health prevention programs: analysis of insights from Australian stakeholders
Source: Front Public Health. 2024 May 3;12:1389739. doi: 10.3389/fpubh.2024.1389739 (PMC11100413; doi:10.3389/fpubh.2024.1389739)
Supplement: Supplementary file 2 [file Data_Sheet_2.PDF]

Appendix 2. Semi-Structured Interview Guide: Digital Health Prevention Programs for Adolescents Relating to Lifestyle Health Behaviours

Interviewer to briefly introduce themselves

Inform the need to audio record the session and remind interviewees the session will not be video recorded so they can keep their cameras on if they would like to and feel comfortable to do so.

Explain that they can withdraw from the interview at any time; however, any contribution to the discussion prior to withdrawal cannot be removed from the recording or written transcript.

<u>Introductions</u>

Explain the purpose of the project and bring up respectful discussion

<u>Main purpose</u>

Explain purpose of project

We are researchers from The University of Sydney conducting research into digital health prevention programs for adolescents relating to lifestyle health behaviours (physical activity, diet, screen time, sleep, body image etc). National frameworks such as the National Preventive Health Strategy and National Action Plan for Young People recommend digital health strategies, and we are aiming to align our research within these. We have conducted a stakeholder mapping process to identify key stakeholders in lifestyle health promotion for adolescents (13-18 years), of which your organisation has been identified. We are seeking to understand the perspectives of key stakeholders who may influence the success and/or implementation of digital health prevention programs for adolescents relating to lifestyle health behaviours.

'Digital health prevention programs' refers to using a range of technologies (mobile health and applications, telehealth, wearable devices, and artificial intelligence) to protect, promote and sustain the population's health.

'Prevention' refers to decreasing the risk, chance or likelihood of an individual developing obesity or mental illness.

The interview will take a maximum of 45 minutes of your time.

<u>Questions for health/education organisations:</u>

1. What does 'digital health' mean to you?
   <u>Prompt:</u> What digital modalities do you think are most useful for health prevention?
2. What do you think the most important health prevention messages are for adolescents?

Initiatives are defined as any health services or programs that are run routinely or periodically or health resources that are publicly available

3. Within your organisation, can you think of any adolescent-specific health prevention initiatives that are currently available and what are they?
   <u>Prompt:</u> If yes, what is the current reach/uptake of these initiatives?
   <u>Prompt:</u> Do you think that a digital health prevention program would be complementary to any existing initiatives within your organisation? Why/Why not?
   <u>Prompt:</u> How well do you think your organisation is doing with this initiative? Do you have any data on this that you can share?

Reach

4. How many adolescents do you think are currently reached by health prevention/digital health prevention programs?
5. What percentage of adolescents do you believe can be reached through a digital health prevention program?
6. Do you think adolescents who come through your organisation would sign up for a digital health prevention program of their own accord?
7. What do you think would be the best way(s) to get adolescents to sign up to a digital health prevention program?
   <u>Prompt:</u> Flyers in waiting room, presented by staff, newsletter distribution, parent/guardians, social media, as part of school curriculum, in health education classes

Effectiveness

8. Would it be feasible within your organisation to collect data on outcomes to understand whether a digital health prevention program was effective?

Adoption

9. Do you think there would be support within your organisation for a digital health prevention program for adolescents? Why/why not?
10. Do you think staff within your organisation would offer this sort of program to adolescents who come through? Why/why not?
    Prompt: What sort of training or staff support do you think would be needed to implement such a program?
11. What could be done to improve the uptake of a digital health prevention program for adolescents?

Implementation

12. What do you think are the greatest strengths your organisation has for implementing a digital health prevention program for adolescents?
13. What do you forecast to be the biggest barriers or challenges of implementing a digital health prevention program for adolescents?
    Prompt: What resources do you think your organisation would need to be able to support the implementation of digital health prevention programs?

Maintenance

14. How long do you think a digital health prevention program for adolescents should run for?
    Prompt: How often do you think a program such as this should be updated?
    Prompt: Do you think that adolescents should be able to access these programs more than once?
    Prompt: Do you think your organisation would be willing to trial or pilot a digital health prevention program? Why/why not?
15. What systems or support would your organisation need to collect data beyond the research phase?

Questions for youth advocacy groups:

1. What does 'digital health' mean to you?
   Prompt: What digital modalities do you think are most useful for health prevention?
2. What do you think the most important health prevention messages are for adolescents?

Initiatives are defined as any health services or programs that are run routinely or periodically or health resources that are publicly available

3. Can you think of any adolescent-specific health prevention initiatives that are currently available? If yes, what are they?
   Prompt: Do you think that a digital health prevention program would be complementary to any existing health prevention initiatives you are currently running? (e.g. in-person workshops) Why/Why not?
4. Prompt: How well do you think your organisation is doing with this initiative? Do you have any data on this that you can share?

Reach

5. How many adolescents do you think are currently reached by health prevention/digital health prevention programs?
6. What percentage of adolescents do you believe can be reached through a digital health prevention program?
7. Do you think adolescents would sign up for a digital health prevention program of their own accord? Why/why not?
8. What do you think would be the best way(s) to get adolescents to sign up to a digital health prevention program?
   Prompt: Flyers, presented by staff, newsletter distribution, parent/guardians, social media

Effectiveness

9. Are there any ways that your group would be able to assess whether a digital health prevention program is effective?

10. What kind of youth involvement do you think is necessary for a digital health prevention program to be effective?

Adoption

11. Do you think there would be support for a digital health prevention program from adolescents? Why/why not?
12. Do you think staff within your organisation would suggest this sort of program to adolescents who come through? Why/why not?
13. What kind of youth consultation could be done to improve the uptake of a digital health promotion program for adolescents?

Implementation

14. What do you think are the greatest strengths your organisation has for implementing a digital health prevention program for adolescents?
15. What do you forecast to be the biggest barriers of implementing a digital health prevention program for adolescents in either healthcare or community settings?

Maintenance

16. How long do you think a digital health prevention program for adolescents should run for?
    Prompt: How often do you think a program such as this should be updated?
    Prompt: Do you think that adolescents should be able to access these programs more than once?
    Prompt: Do you think your organisation would be willing to trial or pilot a digital health prevention program? Why/why not?
17. What kind of youth involvement strategies should be in place to maintain the relevance and currency of digital health prevention programs?
